# Relationship between Swim Bladder Morphology and Hearing Abilities–A Case Study on Asian and African Cichlids

**DOI:** 10.1371/journal.pone.0042292

**Published:** 2012-08-07

**Authors:** Tanja Schulz-Mirbach, Brian Metscher, Friedrich Ladich

**Affiliations:** 1 Department of Behavioral Biology, University of Vienna, Vienna, Austria; 2 Department of Theoretical Biology, University of Vienna, Vienna, Austria; National Institutes of Health/NICHD, United States of America

## Abstract

**Background:**

Several teleost species have evolved anterior extensions of the swim bladder which come close to or directly contact the inner ears. A few comparative studies have shown that these morphological specializations may enhance hearing abilities. This study investigates the diversity of swim bladder morphology in four Asian and African cichlid species and analyzes how this diversity affects their hearing sensitivity.

**Methodology/Principal Findings:**

We studied swim bladder morphology by dissections and by making 3D reconstructions from high-resolution microCT scans. The auditory sensitivity was determined in terms of sound pressure levels (SPL) and particle acceleration levels (PAL) using the auditory evoked potential (AEP) recording technique. The swim bladders in *Hemichromis guttatus* and *Steatocranus tinanti* lacked anterior extensions and the swim bladder was considerably small in the latter species. In contrast, *Paratilapia polleni* and especially *Etroplus maculatus* possessed anterior extensions bringing the swim bladder close to the inner ears. All species were able to detect frequencies up to 3 kHz (SPL) except *S. tinanti* which only responded to frequencies up to 0.7 kHz. *P. polleni* and *E. maculatus* showed significantly higher auditory sensitivities at 0.5 and 1 kHz than the two species lacking anterior swim bladder extensions. The highest auditory sensitivities were found in *E. maculatus,* which possessed the most intimate swim bladder-inner ear relationship (maximum sensitivity 66 dB re 1 µPa at 0.5 kHz).

**Conclusions:**

Our results indicate that anterior swim bladder extensions seem to improve mean absolute auditory sensitivities by 21–42 dB (SPLs) and 21–36 dB (PALs) between 0.5 and 1 kHz. Besides anterior extensions, the size of the swim bladder appears to be an important factor for extending the detectable frequency range (up to 3 kHz).

## Introduction

In fishes that possess swim bladders or other gas filled cavities in proximity to the inner ears, sound can stimulate the inner ears in two ways. In the direct stimulation pathway a sound source leads to the lagged movement of the denser otolith relative to the fish’s body and the sensory epithelium. Thus the otolith acts as an accelerometer in the inner ear that stimulates the hair cells of the sensory epithelium [1]–[2]. In the second pathway the swim bladder acts as pressure-to-displacement transducer. When the swim bladder oscillates in a sound field, it may transmit energy to the inner ear, which again results in otolith motion relative to the sensory epithelium. This mode of stimulation is thought only to be of importance when the swim bladder and the inner ear are very close to one another or when there is some acoustic coupling between the swim bladder and the inner ear [3]. Fishes that primarily hear via direct stimulation tend to have a narrower hearing bandwidth and poorer hearing sensitivity than taxa possessing hearing specializations (e.g., [4]–[5]).

Several species that belong to different teleost families and orders have evolved anterior swim bladder extensions coming close to or contacting the inner ears leading to improved hearing abilities (for an overview see [6]). Despite the high diversity of the swim bladder morphology in teleosts, comparative studies on the effects of different swim bladder morphology on hearing abilities are rare (e.g., [7]). These few studies also show that specializations may have different effects in different species. In squirrelfishes (family Holocentridae) intimate swim bladder-inner ear connections result in an expansion of the hearing bandwidth and in higher auditory sensitivities as compared with species lacking swim bladder extensions or possessing short extensions not contacting the skull [8]–[9]. In contrast, drums or croakers (family Sciaenidae) having anterior swim bladder extensions display an expanded hearing bandwidth but show similar auditory sensitivities to species lacking extensions [10].

Cichlids provide a good example to investigate effects of swim bladder morphology on hearing abilities because this species-rich family displays a high diversity in their swim bladder morphology. This diversity ranges from swim bladders which are small or completely absent in some species of the genus *Steatocranus*, to species possessing large swim bladders without extensions, to species in which the swim bladder contacts the inner ears via anterior projections, as in members of the Malagasy subfamily Etroplinae [6], [11]–[12]. Despite their swim bladder diversity, cichlids have not been investigated in detail with regard to either swim bladder morphology, hearing abilities, or the effects of different swim bladder morphology on hearing abilities [6].

Our study aimed to improve the understanding of the effects of different swim bladder morphology (from small and without anterior extensions to highly specialized) on hearing abilities in terms of hearing bandwidth and auditory sensitivities. We asked the question of whether cichlid species with anterior swim bladder extensions possess higher auditory sensitivities and/or a broader hearing bandwidth than species without such extensions. We thus aimed to test the hypothesis that a close swim bladder-inner ear association results in an improved sensitivity above several hundred Hertz and/or the ability of fishes to detect higher frequencies.

## Materials and Methods

### Study Animals

We chose four cichlid species displaying either (i) small swim bladders (*Steatocranus tinanti*, Slender lion head cichlid, Pseudocrenilabrinae), (ii) large swim bladders without contact to the inner ear (*Hemichromis guttatus*, Jewel cichlid, Pseudocrenilabrinae), or (iii) swim bladders with anterior extensions coming close to the inner ears (*Paratilapia polleni*, Ptychochrominae; *Etroplus maculatus*, Orange chromide, Etroplinae). All four species were investigated with regard to their swim bladder morphology and hearing sensitivity (Table 1). When possible, we used the same specimens of a species for morphological and auditory measurements; when this was not possible, we used individuals from the same source. Fishes originated from local fish suppliers and were transferred to the University of Vienna in August/September 2011 and January 2012 for the auditory analyses. Animals were kept in 98-to 245-l aquaria, which were equipped with a sand bottom, halved flower pots as hiding places, and external filters. No internal filters or air stones were used in order to create a quiet acoustic environment for the test fish. Fishes were kept under a 12∶12 h L:D cycle at 25±1°C and were fed once daily with commercial flake food and red blood worms. Fishes were given a habituation period of at least one week prior to the auditory experiments. All hearing experiments were performed with the permission of the Austrian Federal Ministry of Science and Research (permit number GZ 66.006/0023-II/10b/2008).

**Table 1:** Number of specimens, range of standard length (SL) and fixative/staining method used for the investigation of swim bladder morphology as well as number of specimens, range of standard length and body weight (BW) used for auditory measurements.

	Swim bladder preparation			microCT and 3D reconstructions			Auditory measurements		
Species	*N*	SL (mm)	Fixative	*N*	SL (mm)	Fixative/Staining	*N*	SL (mm)	BW (g)
*Steatocranus tinanti*	6	42–63	10% F (4) 70% EtOH (2)	–	–	–	8	42–65	1.0–4.6
*Hemichromis guttatus*	4	42–55	10% F (3) 70% EtOH (1)	–	–	–	7	44–57	2.3–5.2
*Paratilapia polleni*	5	47–75	10% F (4) 70% EtOH (1)	3	47–54	10% F I_2_KI	5	47–86	3.4–25.2
*Etroplus maculatus*	4	34–41	10% F (4)	4	33–41	10% F I_2_KI	8	33–41	1.3–2.8

EtOH, ethanol; F, formalin; I_2_KI, Lugol solution (2.5% potassium iodide (KI), 1.25% iodine metal (I_2_) in water. Numbers in parentheses indicate number of specimens subjected to the respective treatment.

### Swim Bladder Morphology and Swim Bladder-inner Ear Relationship

#### Preparations and drawings

Ventrolateral dissections of the swim bladder and inner ears were performed for four to six specimens (including the individuals previously used for microCT, see below) of each species. Dissecting microscopes (Wild M7 and Wild M5, Wild Heerbrugg Ltd, Heerbrugg, Switzerland) equipped with a camera lucida were used for preparations and drawings. Preparation of the individuals subjected to microCT scans showed that otoliths were not affected by the formalin fixation.

Light microscopic images were taken using a Leica M165C stereomicroscope with a DFC 290 camera, applying the multifocus option (extended focus imaging) of ImageAccess Standard 8 (Imagic AG, Glattbrugg, Switzerland).

#### MicroCT and 3D reconstructions

For a detailed study of the swim bladder-inner ear relationship in *P. polleni* and *E. maculatus*, we performed high-resolution X-ray microtomographic (microCT) scans ([13]; details in Table 2). Specimens were fixed in 10% formalin at 4°C for up to two days and then stained in a near-isotonic Lugol solution (2.5% potassium iodide (KI), 1.25% iodine metal (I_2_) in water; [14]) at room temperature for a few days to one week in order to enhance tissue contrast.

**Table 2:** Overview of specimens of *P. polleni* and *E. maculatus* subjected to microCT imaging including details about voxel size, image resolution, and number of images used for the 3D reconstructions.

		*Paratilapia polleni*			*Etroplus maculatus*			
	Specimen no.	1	2	3	1	2	3	4
Fish data	SL (mm)	47	54	49	36	40	41	33
	BW (g)	3.4	5.4	3.6	1.7	2.6	2.5	1.1
Whole fish scan	Voxel size (µm)	x = 28 y = 28 z = 25	x = 42 y = 42 z = 33	–	–	x = 33 y = 33 z = 25	–	–
	Image resolution (pixels/inch)	900	600	–	–	770	–	–
	Number of images	1799	1764	–	–	1742	–	–
Close-up scan	Voxel size (µm)	–	x = 15 y = 15 z = 15	x = 15 y = 15 z = 15	x = 24 y = 24 z = 24	x = 15 y = 15 z = 10	x = 15 y = 15 z = 10	x = 9.66 y = 9.66 z = 9.66
	Image resolution (pixels/inch)	–	1719	1719	1067	1715	1715	150
	Number of images	–	981	980	495	996	996	497

BW, body weight; SL, standard length.

A SkyScan 1174 scanner employing a 50 keV/40 W tungsten X-ray source and a 1.3 megapixel CCD camera was used. The images were scanned using isotropic resolution, were reconstructed without binning, and were finally stored as BMP image stacks. A ring-artifact-reduction utility was engaged (setting 7–10) during reconstruction for all the images. For one specimen of *E. maculatus* (specimen no. 4 in Table 2) a high-resolution scan of the inner ear and the anterior most part of the swim bladder was performed with a MicroXCT from Xradia Inc., Pleasanton, CA (www.xradia.com). This scanner uses a tungsten X-ray source with an anode voltage setting of 80 kV at 8 W, and using 2-fold magnification with an exposure time of 30 seconds for projection images every 0.25°.

Prior to 3D rendering, image stacks were edited in AdobePhotoshop® CS2. Images were reduced from 16 bit to 8 bit grayscale, cropped, and, if necessary, resolution was reduced to a minimum image resolution of 600 pixels per inch for whole-fish scans and to a minimum image resolution of 1,715 pixels per inch for close-up scans (in *E. maculatus* 1 1,067 pixels per inch represents the original image resolution; for further details see Table 2). Close-up scans covered the region from the eyes to approximately the anterior third of the swim bladder.

3D renderings of otoliths and swim bladders were performed in AMIRA® v. 5.4.0 (Visage Imaging GmbH, Berlin, Germany). A threshold-based segmentation was applied for labeling the structures and, if necessary, this labeling was refined or corrected using the brush tool. In the case of the otoliths and the swim bladder horns, every image was labeled. For the reconstruction of the posterior and middle parts of the swim bladder, initially every 10^th^ to 20^th^ image was labeled, with subsequent interpolation of structures on intervening images, followed by check and–if required–correction of segmentation results.

Subsequently, every otolith type as well as the swim bladder were separated from the ‘master’ LabelField file into single LabelFields and saved as separate files. The new LabelField for the swim bladder was reduced in resolution applying the Resample module. Surface rendering was performed with the SurfaceGen module. This was followed by the smoothing of surfaces using the SmoothSurface module (40 iterations for the swim bladder and 20 iterations for each ototlih type; unconstrained smoothing).

In order to visualize the in-situ position of the swim bladder and the otoliths, the whole fish was displayed using the volume rendering tool (Volren settings used: mode  =  maximum intensity projection, MIP; alpha  = 1; color  =  grey.am). Finally, an overlay of the volume rendered whole fish and the 3D rendered otoliths and the swim bladder was created.

### Auditory Sensitivity Measurements

Auditory thresholds were determined by applying the auditory evoked potential (AEP) recording technique [15]–[17]. The AEP technique records far-field potentials in response to sound stimuli of the whole auditory pathway from the inner ear up to midbrain nuclei [18].

In order to reduce muscle noise, the test subjects were immobilized with Flaxedil (gallamine triethiodide; Sigma Aldrich Handels GmbH, Vienna, Austria) at mean concentrations of 7.4, 27.2, 17.9, or 7.2 µg*g^–1^ body weight for *S. tinanti*, *H. guttatus*, *P. polleni*, and *E. maculatus*, respectively. All auditory measurements were carried out in an oval-shaped plastic tub (diameter 45×30 cm, water depth 12 cm, 0.5 cm layer of sand), which was lined inside with acoustically absorbent material (air-filled packing wrap) to minimize resonances and reflections (see Figure 1 in [19]). The set-up for auditory sensitivity measurements, the presentation of sound stimuli, and the determination of thresholds followed the detailed description given by [20]. Temperature in the tank was controlled at 25±1°C. In addition, room temperature was kept constant at about 25°C. *S. tinanti* were tested at nine different frequencies from 0.1 up to 1 kHz (including 0.9 kHz); *H. guttatus*, *P. polleni*, and *E. maculatus* were tested at seven (*P. polleni*; *E. maculatus*) or eight (*H. guttatus*) different frequencies ranging from 0.1 up to 3 kHz.

**Figure 1:**
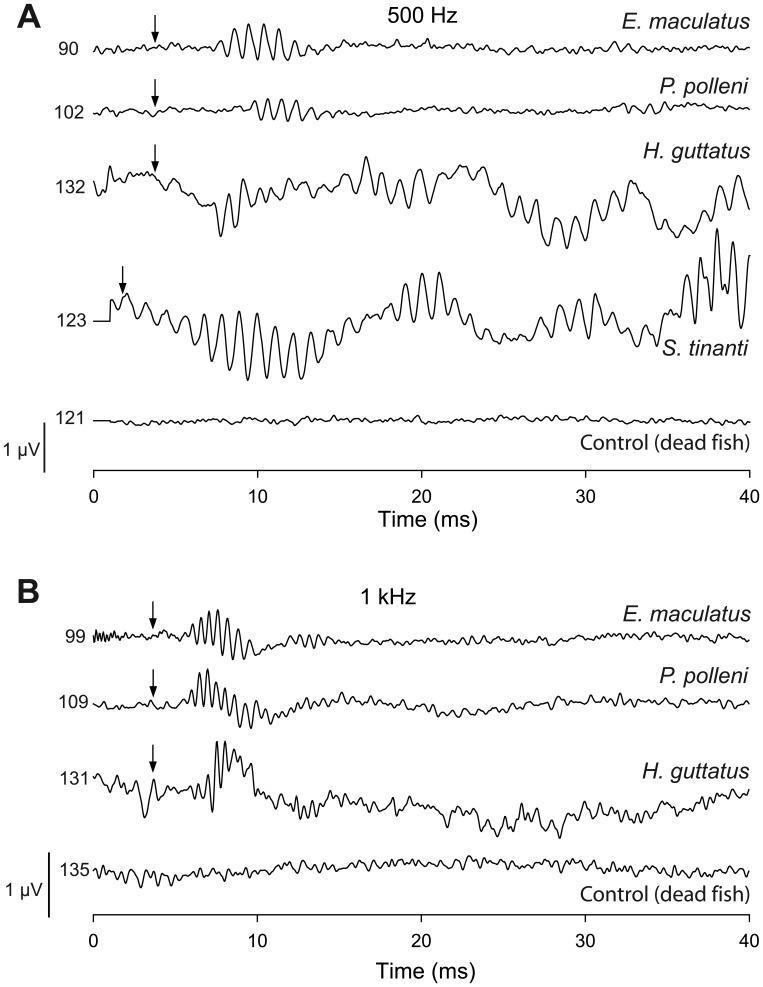
Representative AEP waveforms in response to 0.5 and 1 kHz tone bursts and control recording of a dead fish. AEPs are shown 20 dB above the mean auditory threshold of each species (see Table 3A). Numbers indicate SPLs (dB re 1 µPa). Arrows indicate the stimulus onset.

In order to make sure that AEPs were not artifacts, we tested our system with dead fishes and with no fish in the set-up. No responses were obtained from dead fishes (Fig. 1).

### Particle Acceleration Measurements

In addition to sound pressure levels (SPL), we determined particle acceleration levels (PAL) at thresholds because fish species lacking hearing specializations lack sound pressure sensitivity [3], [21].

In order to compare SPLs and PALs for frequencies up to 1 kHz, a calibrated underwater miniature acoustic pressure-acceleration (p-a) sensor (S/N 2007-001, Applied Physical Sciences Corp., Groton, CT, USA; frequency bandwidth: 20 Hz to 2 kHz; sensitivity: −137.6 dB re 1 V/µm/s^2^) was placed at the fish’s position in the test tub. PALs at all stimulus frequencies and at hearing threshold levels of the fish were determined with the acceleration sensor oriented in all three orthogonal directions. In consistence with previous studies [7], [21]–[22], the x-axis was considered to be anterior–posterior along each subject’s body, the y-axis was considered to be lateral (right–left) relative to the subject, and the z-axis to be vertical (i.e., up-down) relative to the subject. This approach yielded simultaneous measurements of sound pressure and particle acceleration in all three directions over the entire stimulus range. SPLs were calculated in dB RMS re 1 µPa and PALs in dB RMS re 1 µm/s^2^ (Table 3). These are the international units for sound pressure and particle acceleration according to ISO standards [23].

**Table 3:** Sound pressure level and particle acceleration levels in the three orthogonal Cartesian directions and for PAL of the three axes combined at each test frequency.

Frequency (kHz)	SPL	PAL vert	PAL rc	PAL lat	PAL comb
0.1	100	59	55	40	61
0.2	98	58	51	46	59
0.3	101	65	58	54	66
0.5	100	64	49	41	64
0.6	100	62	48	41	62
0.7	100	61	48	44	61
0.8	98	60	47	47	60
1.0	100	62	54	54	63

SPL-sound pressure level (dB re 1 µPa), PAL-particle acceleration level (dB re 1 µm/s^2^) in the vertical (vert), rostrocaudal (rc), and lateral (lat) axis; PAL comb-PAL combined of the three directions (magnitude *sensu* Casper and Mann (2006) [22] and Wysocki et al. (2009) [x]. The PAL comb was calculated based on the particle acceleration of each axis in µm/s^2^ as 20*log(sqrt(x^2^+y^2^+z^2^)).

### Ambient Noise Measurements

The spectral level of the laboratory ambient noise was measured and calculated following the methods described in [17] and [21].

### Statistical Analyses

A repeated measures design was applied for analyzing potential differences between audiograms (see also [7]). Auditory thresholds expressed in terms of SPLs or PALs obtained from auditory measurements were analyzed by calculating a full factorial general linear model with ‘frequency’ as repeated measures (rmGLM), ‘thresholds’ as dependent variable, and ‘species’ as fixed factor. Because the detectable frequency range of *S. tinanti* was distinctly smaller than in the other three species, we performed two analyses, one with all four species from 0.1 up to 0.5 kHz, and a second one without *S. tinanti* focusing on the three species across the entire range of frequencies tested. We analyzed thresholds at frequencies of 0.1, 0.3, 0.5, 0.8, 1, 2, and 3 kHz based on SPLs and at frequencies of 0.1, 0.3, 0.5, 0.8, and 1 kHz based on PALs. All statistical analyses were conducted in PASW 18.0 (SPSS Inc., Chicago, IL, USA). Differences between species were tested applying post-hoc tests (Tukey HSD) with α = 0.05.

## Results

### Swim Bladder Morphology and Swim Bladder-inner Ear Relationship

All four species possessed a swim bladder with a transverse diaphragm (including a sphincter) dividing the organ into an anterior and a posterior chamber. Except in *E. maculatus*, the swim bladder of all species had a more opaque and thicker walled anterior chamber and a translucent and very thin walled posterior chamber. In *E. maculatus*, the whole swim bladder was thick-walled with a silvery whitish appearance.

In the following, the inner ear-swim bladder distance is defined as the distance between the posterior most region of the ear to the anterior most part of the swim bladder. In species with extensions, this is the distance between the anterior most part of the left or right swim bladder extension to the posterior most part of the left or right ear.

#### Vestigial swim bladder

**Figure 2:**
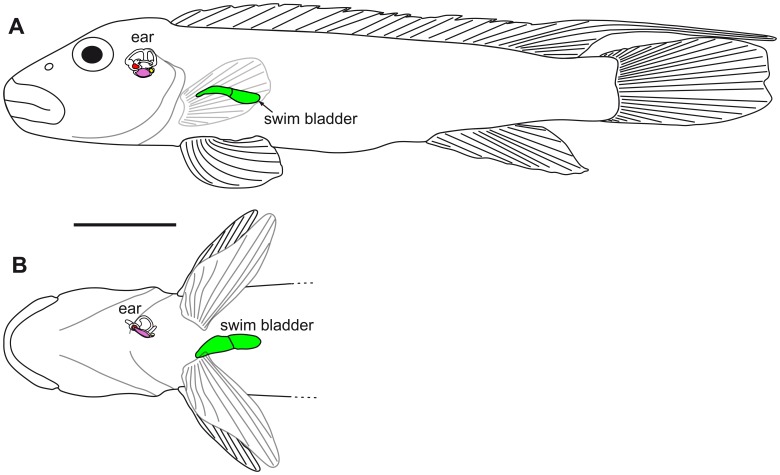
Swim bladder and inner ear of *S. tinanti* in (A) lateral and (B) ventral views. The swim bladder is small and distinctly away from the inner ear. The swim bladder is shown in green; the otoliths of the inner are shown in red (lapillus  =  utricular otolith), pink (sagitta  =  saccular otolith), and yellow (asteriscus  =  lagenar otolith). Scale bar  = 1 cm.

*Steatocranus tinanti* showed a small swim bladder without anterior extensions located in the anterior body cavity distinctly away from the inner ears (Fig. 2). In some specimens, the anterior chamber of the swim bladder was asymmetrically developed (Fig. 2B). One out of the six dissected specimens displayed a very small swim bladder whereas another individual possessed a slightly larger swim bladder situated in the middle of the body cavity.

#### Swim bladder without anterior extensions

**Figure 3:**
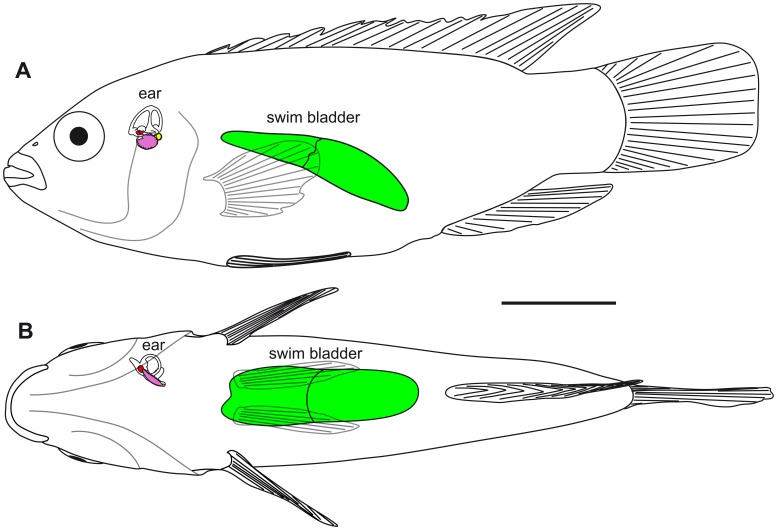
Swim bladder and inner ear of *H. guttatus* in (A) lateral and (B) ventral views. The swim bladder is ‘normal’ sized without contact to the inner ear. The swim bladder is shown in green; the otoliths of the inner are shown in red (lapillus), pink (sagitta), and yellow (asteriscus). Scale bar  = 1 cm.

*H. guttatus* showed a large swim bladder with two small slightly asymmetrical anterior bulges but without anterior extensions distinctly away from the inner ears (Fig. 3).

#### Swim bladder with anterior extensions

**Figure 4:**
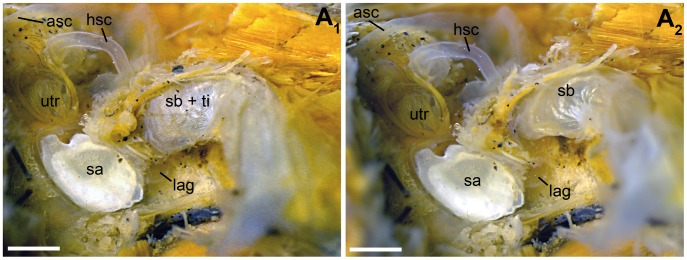
Ventrolateral view of the left inner ear and the anterior most part of the left swim bladder horn in *P. polleni* (specimen 1, **Table 2** **).** A tissue sheath envelopes the swim bladder horn (A_1_). In (A_2_) the swim bladder horn is shown after the removal of the tissue sheath. asc, anterior semicircular canal; hsc, horizontal semicircular canal; lag, lagena; sa, sagitta; sb, swim bladder horn; sb + ti, swim bladder horn with enveloping tissue sheath; utr, utricle. Scale bars  = 1 mm.

**Figure 5:**
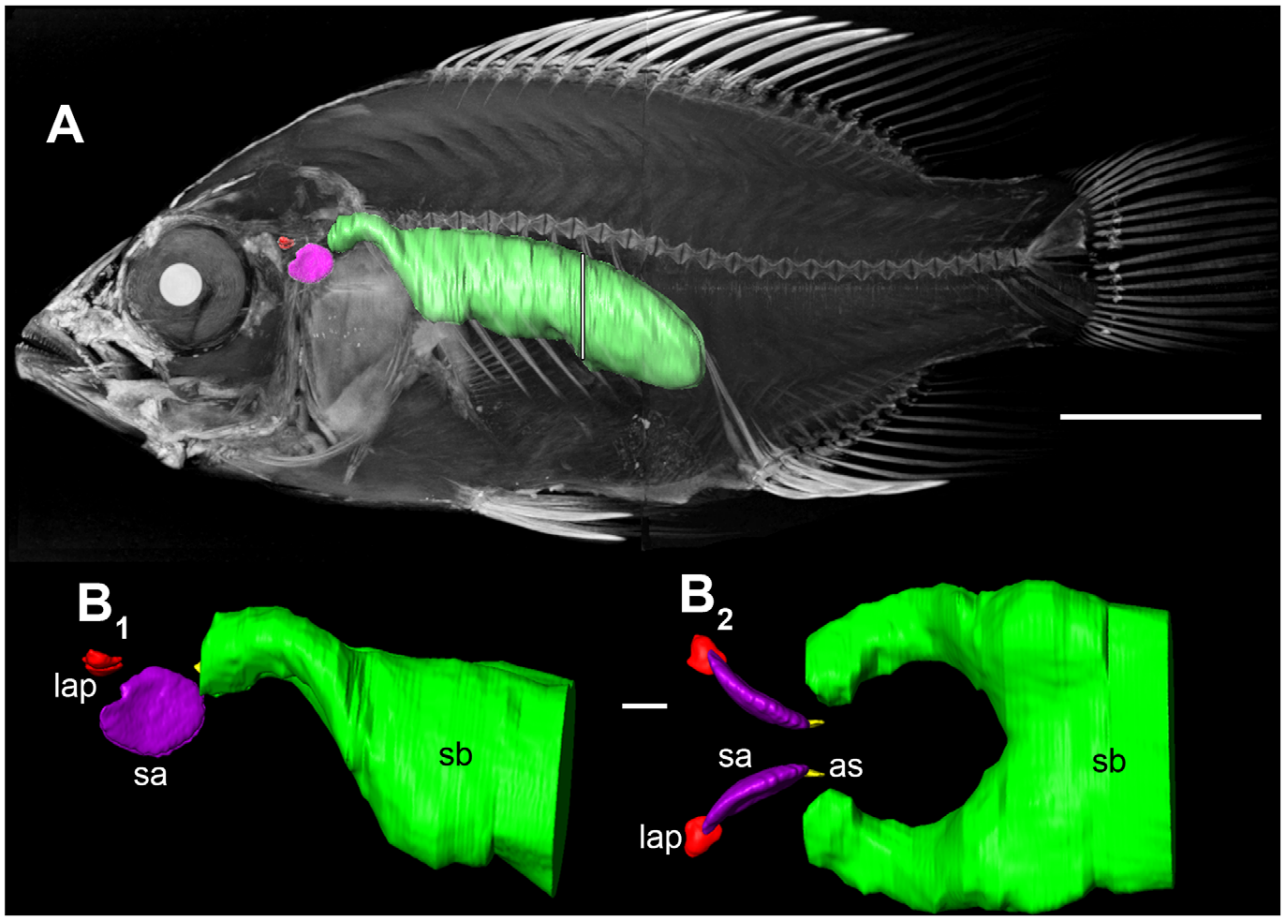
3D reconstructions of the swim bladder and the otoliths of *P. polleni* displaying the anterior projections of the swim bladder to the inner ear. (**A**) Volume rendering based on the reconstruction of the whole fish (specimen 2, see Table 2) in lateral view with the swim bladder (green) and the otoliths lapillus (red), sagitta (pink), and asteriscus (yellow) superimposed. The white line indicates the position of the septum. (**B**) Reconstructions of the close-up scan of the otoliths and the anterior portion of the swim bladder in (**B_1_**) lateral and (**B_2_**) ventral views. The swim bladder horns come close to the asteriscus. as, asteriscus; lap, lapillus; sa, sagitta; sb, swim bladder. Scale bars: (**A**) 1 cm, (**B**) 1 mm.

**Figure 6:**
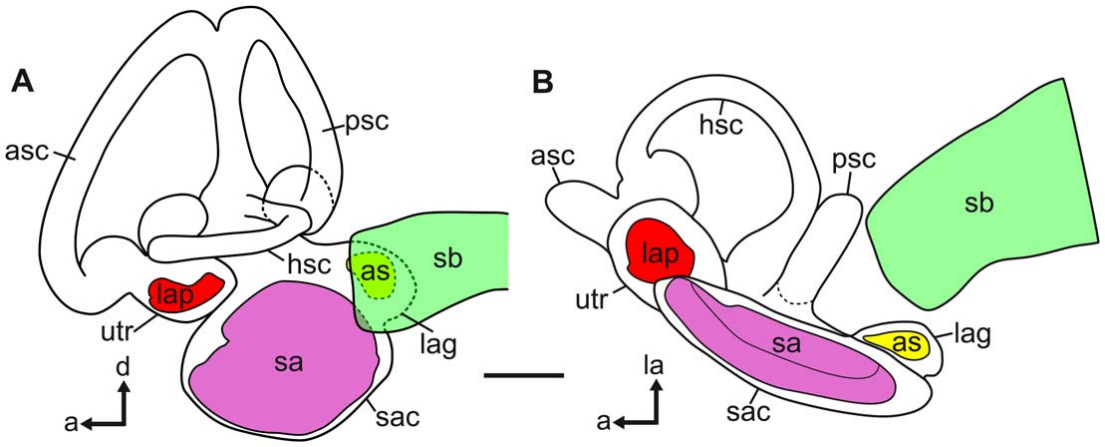
Swim bladder and inner ear of *P. polleni* in (A) lateral and (B) ventral views. The swim bladder horn comes close to the lagena and its otolith, the asteriscus (see also Figure 5). a, anterior; as, asteriscus; asc, anterior semicircular canal; d, dorsal; hsc, horizontal semicircular canal; la, lateral; lap, lapillus; lag, lagena; psc, posterior semicircular canal; sa, sagitta; sac, sacculus; sb, swim bladder; utr, utricle. Scale bar  = 1 mm.

In *P. polleni*, the two tube-like swim bladder horns curved dorsally and then slightly medially approaching the posterior skull. The horns were enveloped by extensions of the septum transversum and the parietal peritoneum that connected the horns to the exoccipital foramina (Fig. 4). The swim bladder horns came close to the inner ears (Figs. 5,6) but did neither penetrate the skull nor did they directly contact the inner ears.

**Figure 7:**
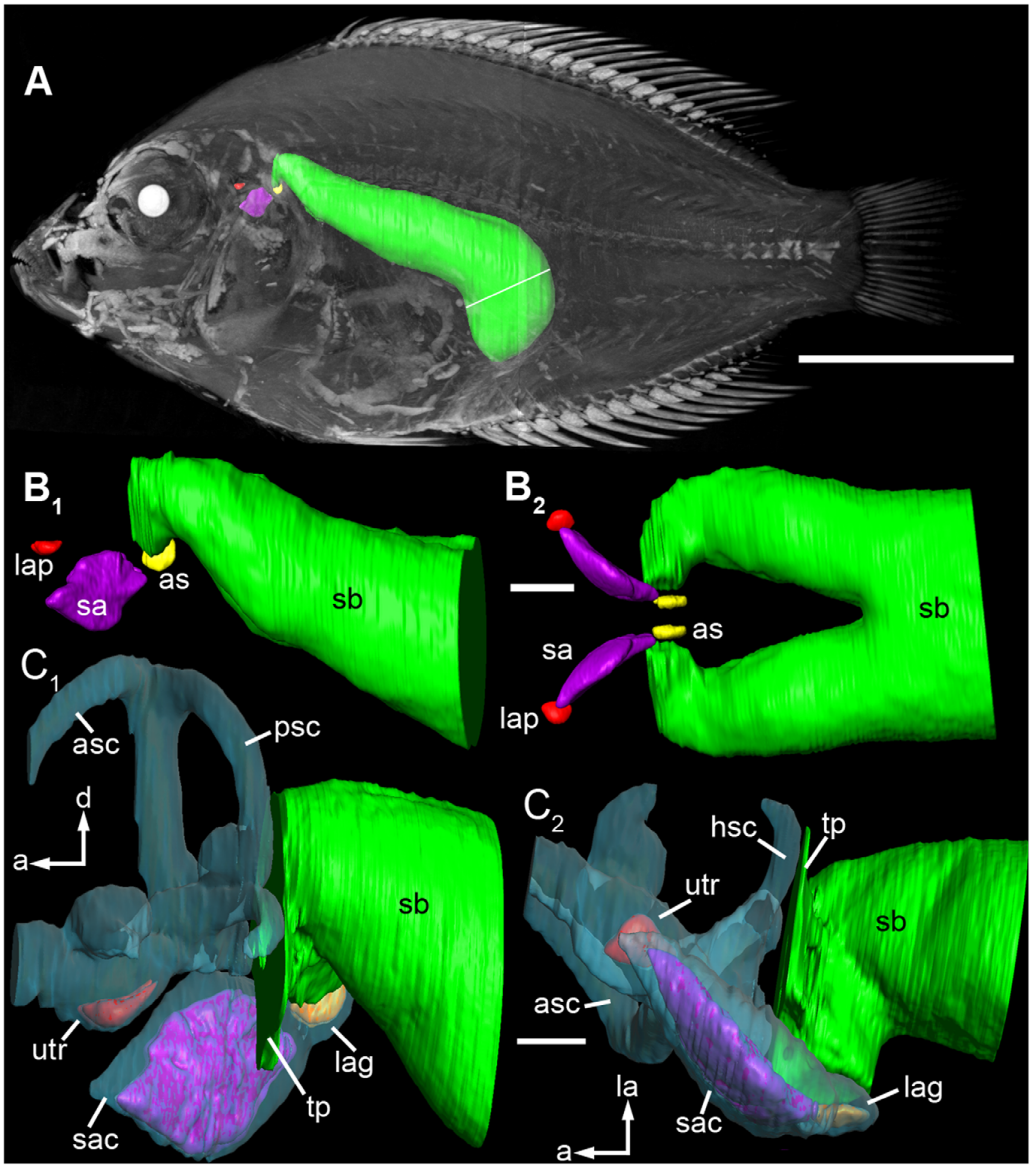
3D reconstructions of the swim bladder and the otoliths of *E. maculatus* displaying the rostral projections of the swim bladder to the inner ear. (**A**) Volume rendering based on the reconstruction of the whole fish (specimen 2, see Table 2) in lateral view with the swim bladder (green) and the otoliths lapillus (red), sagitta (pink), and asteriscus (yellow) superimposed. The white line indicates the position of the septum. (**B**) Reconstructions of the close-up scan of the otoliths and the anterior portion of the swim bladder in (**B_1_**) lateral and (**B_2_**) ventral views. The swim bladder horns come very close to the asteriscus. (**C**) The high-resolution close-up of another individual (specimen 4, Table 2) additionally shows the membranous labyrinth and the tissue pad of the swim bladder horn displaying the close proximity to the lagena and the posterior and horizontal semicircular canals in (**C_1_**) lateral and (**C_2_**) ventral views. Note that anterior and horizontal semicircular canals are incomplete due to the limited scanning field. a, anterior; as, asteriscus; d, dorsal; la, lateral; lap, lapillus; sa, sagitta; sb, swim bladder; tp, tissue pad. Scale bars: (**A**) 1 cm, (**B**) 1 mm, (**C**) 500 µm.

**Figure 8:**
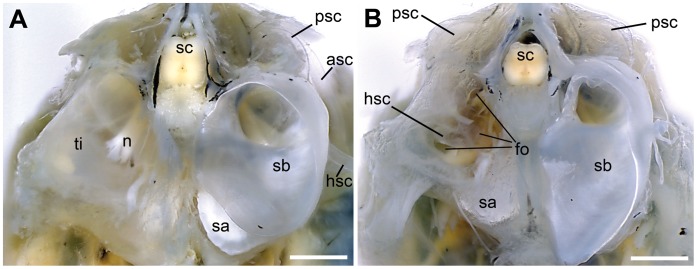
Posterior views of the anterior most swim bladder extension and occipital region of the skull of *E. maculatus*. (**A**) The left swim bladder extension was removed and exposes the tissue covering the foramen in the exoccipital bone (specimen 3 in Table 2). On the right side, the anterior most swim bladder extension and the exposed inner ear can be seen. In another specimen (specimen 4 in Table 2) the swim bladder extension and tissue were both removed in order to expose the foramen (**B**). On the right side, the anterior most swim bladder extension was left in position. The inner ears on both sides are still covered by bone. asc, anterior semicircular canal; fo, exoccipital foramen; hsc, horizontal semicircular canal; n, nerve; psc, posterior semicircular canal; sa, sagitta; sb, swim bladder extension; sc, spinal chord; ti, tissue sheet. Scale bars  = 1 mm.

**Figure 9:**
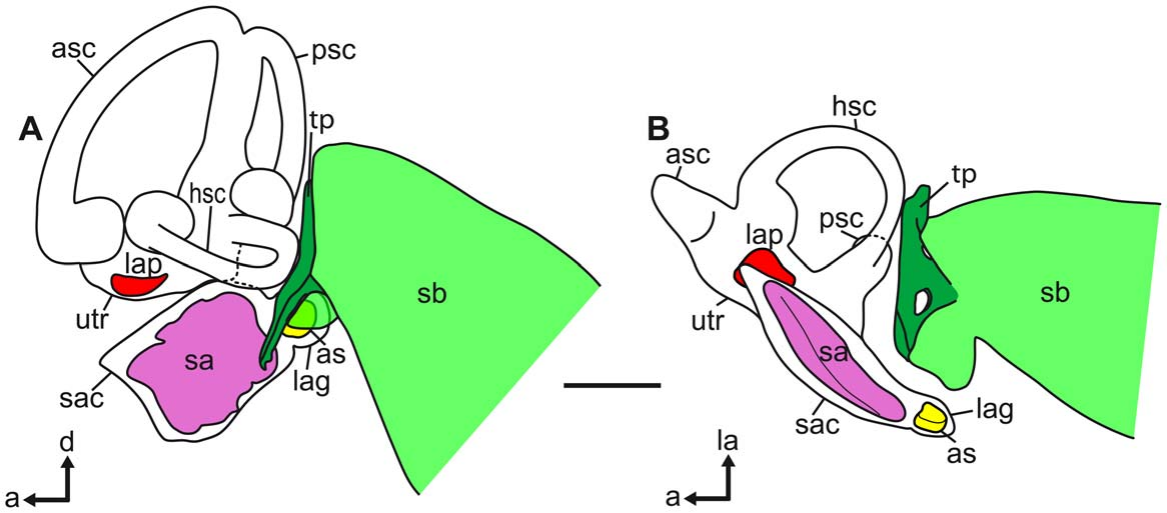
Swim bladder and inner ear of *E. maculatus* in (A) lateral and (B) ventral views. The air-filled part of the swim bladder is shown in shaded green and the tissue pad in dark green. The two white openings in the tissue pad (**B**) are passages for nerves. a, anterior; as, asteriscus; asc, anterior semicircular canal; d, dorsal; hsc, horizontal semicircular canal; la, lateral; lap, lapillus; lag, lagena; psc, posterior semicircular canal; sa, sagitta; sac, sacculus; sb, swim bladder; tp, tissue pad; utr, utricle. Scale bar  = 1 mm.

In *E. maculatus*, the anterior most regions of the swim bladder extensions were made up of two different parts. Firstly, there was an air-filled portion curving medially and coming close to the lagena without however penetrating the bone covering this end organ. This part could be illustrated by the close-up scans (Fig. 7B,C). The second part of the swim bladder extension was a tissue pad enveloping a complex foramen in the exoccipital bone (F. 8B). The contact between the tissue pad of the swim bladder extension and the posterior part of the upper labyrinth (the posterior semicircular canal and the posterior part of the horizontal semicircular canal) was mediated by a tissue sheet spanning in the foramen (Fig. 8A). The tissue pad was identified by dissections (Fig. 9) as well as by the high-resolution close-up microCT scan (Fig. 7C). In contrast to the other three species, the posterior part of the swim bladder in *E. maculatus* was strongly curving ventrally (Fig. 7A).

### Auditory Sensitivities

**Figure 10:**
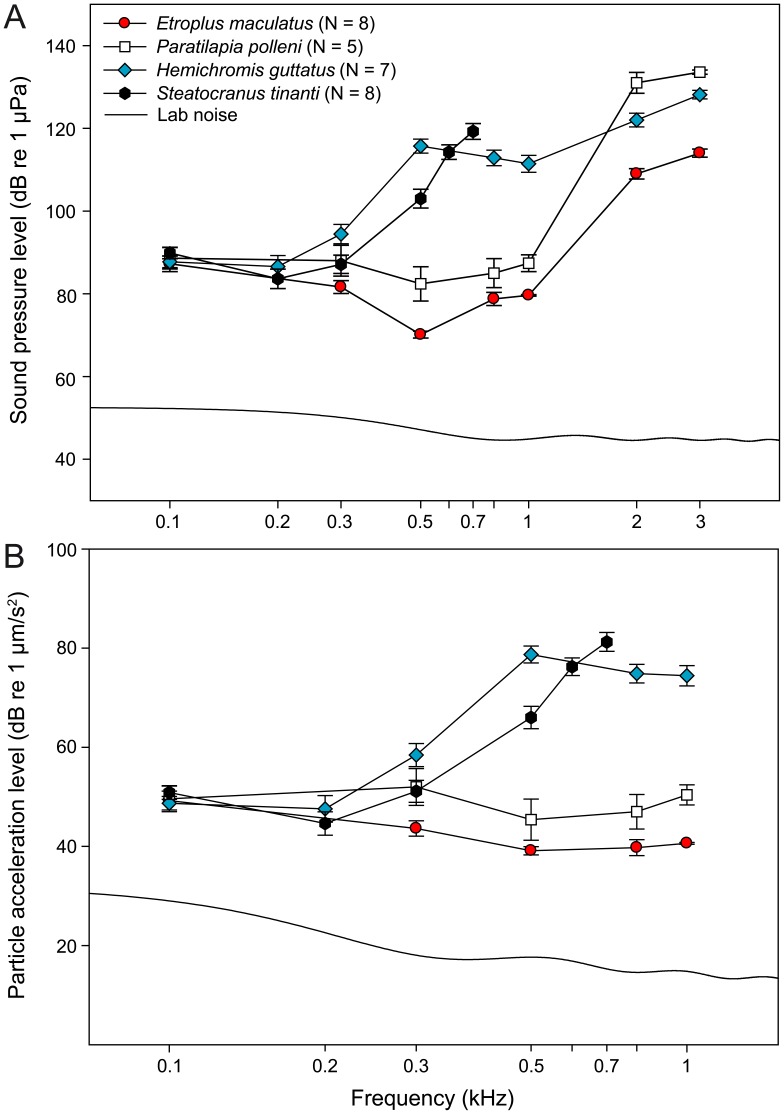
Mean (± s.e.m.) hearing thresholds of the cichlid species investigated. (**A**) Sound pressure level (SPL) audiograms and (**B**) Particle acceleration level (PAL) audiograms. Lab noise, cepstrum-smoothed spectra of laboratory ambient noise.

*H. guttatus, P. polleni*, and *E. maculatus* responded to tone bursts from 0.1 up to 3 kHz whereas in *S. tinanti* no response was detectable above 0.7 kHz (Table 4; Fig. 10). Auditory sensitivities differed significantly between species and sensitivities at the respective frequencies depended significantly on the species tested (Table 5; Fig. 10). Post-hoc tests for the data set with *S. tinanti* (0.1–0.5 kHz) revealed significant differences (*P*≤0.022) between all four species except *H. guttatus* vs. *S. tinanti* (SPL), *E. maculatus* vs. *P. polleni* (PAL), and *H. guttatus* vs. *S. tinanti* (PAL) while the dataset without *S. tinanti* (0.1–3 kHz) showed that the remaining three species differed significantly from each other with regard to SPL (*P*<0.001) and PAL (*P*≤0.001) thresholds. *S. tinanti* and *H. guttatus* showed best auditory sensitivity at 0.2 kHz while *P. polleni* and *E. maculatus* had best auditory sensitivity at 0.5 kHz (Table 4; Fig. 10). All four species displayed similar sensitivity between 0.1 and 0.3 kHz. Above 0.3 kHz, auditory sensitivity in *P. polleni* and *E. maculatus* increased slightly while it decreased steeply in *S. tinanti* and *H. guttatus.* This resulted in sensitivity differences between species of 21–42 dB (SPL) and of 21–36 dB (PAL). Sensitivity was highest in *E. maculatus* at all frequencies above 0.3 kHz. Above 1 kHz, *P. polleni* and *E. maculatus* showed a decrease in sensitivity which was more pronounced in *P. polleni*. Laboratory ambient noise spectral levels (sound pressure and particle acceleration) were at least 20 dB below hearing thresholds (Fig. 10A and B).

**Table 4:** Mean (± s.e.m.) auditory sensitivity of the four cichlid species investigated.

Species	*S tinanti*	*H. guttatus*	*P. polleni*	*E. maculatus*	*S. tinanti*	*H. guttatus*	*P. polleni*	*E. maculatus*
*N*	8	7	5	8	8	7	5	8
Frequency (kHz)	Sound pressure level (dB re 1 µPa)				Particle acceleration level (dB re 1 µm/s^2^)			
0.1	90±1.4	88±1.4	89±2.6	88±1.3	51±1.4	49±1.4	50±2.6	50±1.3
0.2	84±2.4	87±2.7	nm	nm	45±2.4	48±2.7	nm	nm
0.3	87±2.4	94±2.3	88±3.7	82±1.6	51±2.4	58±2.3	52±3.7	44±1.6
0.5	103±2.3	112±3.5	82±4.2	70±0.8	66±2.3	75±3.5	45±4.2	39±0.8
0.6	113±1.3	nm	nm	nm	75±1.3	nm	nm	nm
0.7	118±1.9	nm	nm	nm	80±1.9	nm	nm	nm
0.8	nr	113±1.9	85±3.5	79±1.6	nr	75±1.9	47±3.5	40±1.6
1	nr	111±2.3	87±2.0	80±0.2	nr	74±2.3	50±2.0	41±0.2
2	nm	122±1.7	131±2.4	109±1.2				
3	nm	128±1.0	133±0.5	114±1.0				

*N*, number of specimens; nm, no measurement taken; nr, no response.

**Table 5:** Full factorial general linear models (GLM) using ‘frequency’ as repeated measurements and ‘species’ as fixed factor were calculated for all four species from 0.1 up to 0.5 kHz or with *S. tinanti* removed from the dataset for the full frequency range tested.

		Sound pressure level (dB re 1 µPa)			Particle acceleration level (dB re 1 µm/s^2^)		
Source	df	MS	F	P	MS	F	P
**All species (0.1–0.5 kHz)**							
**Between-subjects effects**							
Species	3	1385.123	31.342	**<0.001**	1150.520	26.033	**<0.001**
Error	24	44.194					
**Within-subject effects**							
Frequency	1	141.280	4,152	0.053	567.617	16.680	**<0.001**
Frequency × Species	3	1322.599	38.866	**<0.001**	1058.075	31.093	**<0.001**
Error	24	34.029			34.029		
**Without ** ***S. tinanti*** ** (0.1–3 kHz)**							
**Between-subjects effects**							
Species	2	5731.730	178.482	**<0.001**	5351.470	174.115	**<0.001**
Error	17	32.114			30.735		
**Within-subject effects**							
Frequency	1	22468.492	1045.975	**<0.001**	345.229	9.547	**0.007**
Frequency × Species	2	388.819	18.101	**<0.001**	1599.012	44.218	**<0.001**
Error	17	21.481			36.162		

Significant *P*-values are bold. MS, mean squares.

## Discussion

We tested the hypothesis that different swim bladder morphology in cichlids affects their hearing sensitivities. We found that species having swim bladder extensions directly or indirectly touching the inner ears showed a distinctly higher auditory sensitivity and/or a broader hearing bandwidth than species lacking these structures. Furthermore, we showed that the size of the swim bladder influenced hearing in addition to presence or absence of anterior swim bladder extensions.

### Diversity of Swim Bladder Morphology among Cichlids

The swim bladder morphology we found in *Paratilapia* and *Etroplus* are in accord with previous findings [11]–[12]. Dehadrai (1959) [11] reported a “tendinous pad” at the anterior end of the swim bladder extensions contacting the inner ear in *Etroplus*. In contrast to our in situ imaging and description of the swim bladder-inner ear relationship this author illustrated the ‘auditory caecum’ ( =  anterior swim bladder extension) separated from the ‘auditory fontanelle’ ( =  exoccipital foramen), making it difficult to understand the anatomical and functional relationship. In addition, the author did not mention the presence of the lagena and thus did not illustrate or describe the proximity of the anterior swim bladder extensions to this end organ. Sparks (2008) [12] described a large exoccipital foramen in *Etroplus* and swim bladder horns in *Paratilapia* that terminate at the skull generally without penetrating it. However, these findings were not illustrated for these two species in detail and the author did not report a tissue sheath enveloping the swim bladder horns in *P. polleni* [12]. Braun and Grande (2008) [6] mentioned a tiny swim bladder in *S. tinanti* without giving any illustrations or details.

Anterior swim bladder extensions are well known from several members of teleost orders such as the clupeiform family Clupeidae (e.g. [24]), the perciform families Sciaenidae [10], [25]–[26], Gerreidae [27], and Chaetodontidae [28]–[29], the beryciform family Holocentridae [30]–[31] and the gadiform family Moridae [32]–[33] (for an overview see [6]). Although some taxa showed complex swim bladder extensions such as *Antimora rostrata* (Moridae) [32] or *Myripristis kuntee* (Holocentridae) [31], there are no other known examples possessing a structure similar to the tissue pad found in *Etroplus*.

So far, the function of the tissue pad is unknown. The pad might enhance the transmission efficiency from the swim bladder to the inner ears. The pad provides a larger contact region to the membranous labyrinth than the air-filled swim bladder extension alone which means that a larger area may transmit the pressure oscillations in the swim bladder wall to the perilymph surrounding the inner ear.

### Auditory Sensitivities in Cichlids

Our data clearly demonstrate that auditory sensitivities at frequencies above 0.3 kHz differ between the investigated cichlid species. The low absolute sensitivity and low maximum detectable frequency (0.7 kHz) in *S. tinanti* indicates that this species lacks sound pressure sensitivity in contrast to the other species measured. For this reason it is necessary to characterize hearing sensitivities in cichlids based on SPLs and PALs. So far, only SPL audiograms of the four cichlid species *Neolamprologus brichardi* [34], *Astronotus ocellatus* [15], [35], *Oreochromis niloticus* [36], and *Tramitichromis intermedius* [37] have been published. Our study is therefore the first one presenting thresholds of cichlids on both bases, which is especially important in species lacking known hearing specializations or in species in which sound pressure sensitivity is unlikely.

### Morphofunctional Correlations

Several morphological features of the swim bladder may influence the hearing abilities in fishes, particularly size, and distance to the inner ears.

In our study *S. tinanti* had a distinctly smaller swim bladder than the other three species and showed a low hearing sensitivity. The adaptation to a rheophilic mode of life in *S. tinanti* [38] may have led to the reduction of the swim bladder size, as seen in many bottom-dwelling fishes, e.g. gobies, sculpins, and some catfishes, which may account for their limited hearing abilities. A comparative study on catfishes demonstrated a significant effect of swim bladder size on hearing abilities [39]. Loricariid and callichthyid catfishes with small encapsulated bladders displayed significantly lower auditory sensitivities at higher frequencies than representatives of families with larger free swim bladders (Ariidae, Doradidae, Pimelodidae, etc.). While the low sensitivity in *S. tinanti* may be an effect of small swim bladder size, the opposite may be assumed for *H. guttatus.* This species is able to detect frequencies up to 3 kHz indicating that the large swim bladder contributes to their high frequency sensitivity despite a lack of a swim bladder-inner ear connection.

The proximity of the swim bladder to the inner ears is likely to play an important role in transmitting sound efficiently to the inner ears. We showed that the closer the swim bladder came to the inner ears the higher the auditory sensitivities at frequencies above 0.3 kHz with exception of two out of eight frequencies in *H. guttattus* and *P. polleni* (at 2 and 3 kHz). Our findings are therefore partly consistent with those in holocentrids and sciaenids.

There exists a discrepancy between holocentrids, sciaenids, and cichlids in the effect of the swim bladder-inner ear relationship on hearing sensitivity. This may partly be due to the fact that the swim bladder morphology and auditory sensitivities have not been investigated in the same study. Moreover, differences in the set-ups for AEP measurements may also make any comparison difficult.

The Holocentridae include species with unspecialized swim bladders (genus *Sargocentron*, formerly *Adioryx*), species with short extensions not contacting the skull (*Holocentrus*) [40] and one genus (*Myripristis*) having long anterior extensions that directly contact the inner ears. These differences in swim bladder morphology have been assumed to account for both differences in hearing bandwidth and auditory sensitivity [8], [30]. *Myripristis kuntee* had a broad hearing bandwidth up to 3 kHz and auditory sensitivities comparable to those measured for goldfish [9] while members of the genus *Sargocentron*, which lack swim bladder specializations responded only up to 1.2 kHz and showed low auditory sensitivities above 0.5 kHz [8]. *Holocentrus adsensionis* showed an intermediate position with regard to hearing bandwidth and auditory sensitivity between *M. kuntee* and *Sargocentron vexillarium*. It is, however, noteworthy that *H. adsensionis* had lower auditory sensitivity (between 0.1 and 0.5 kHz) than another species of the genus *Sargocentron* (*S. xantherythrum*) [30].

In contrast to holocentrids, sciaenids with different swim bladder morphology differed mainly in their hearing bandwidth [7], [10], [25]–[26], but displayed no distinct differences in auditory sensitivity. *Bairdiella chrysoura* and *Cynoscion regalis* that possess a close inner ear-swim bladder connection, detect frequencies up to 4 kHz or, 2 kHz, respectively, whereas *Leiostomus xanthurus* which lacks specializations, only detects frequencies up to 0.7 kHz. While *B. chrysoura*, with the most specialized swim bladder-inner ear contact, displays distinctly higher sensitivities (lowest threshold: 75 dB re 1 µPa at 0.6 kHz), no significant difference in auditory sensitivity was found between *C. regalis* and *L. xanthurus* (lowest threshold for both species: 90 dB re 1 µPa at 0.3 kHz) [10]. Accordingly, a recent study also revealed no significant difference in hearing thresholds in species with (*Cynoscion regalis*, *Cynoscion nebulosus*, *Micropogonias undulatus*) and without swim bladder specializations (*Sciaenops ocellatus*, *Leiostomus xanturus*) [7]. Surprisingly, the authors found that thresholds in *Menticirrhus saxatilis* a species reducing the swim bladder in adults were among the lowest above 0.6 kHz [7].

It should be mentioned that fishes may possess enhanced auditory abilities without obvious morphological specializations. Damselfishes are sound-pressure sensitive and have best hearing sensitivities at 0.5 kHz without any swim bladder-inner ear connection [41]. Furthermore, pronounced auditory sensitivities in the brown meagre *Sciaena umbra* (Sciaenidae) were described without finding any morphological specializations [21]. Similarly, present data reveal that the cichlid *H. guttatus* is sensitive to frequencies up to 3 kHz although it lacks a connection of the swim bladder to the inner ear. In these cases it might be that swim bladder oscillations are transmitted through soft tissue or via the backbone (as suggested for the eel *Anguilla anguilla* [42]) to the inner ears. This assumption could, however, only be tested by conducting swim bladder deflation experiments [43]–[44].

### Evolutionary Considerations

Different selective pressures may act on swim bladder size and the development of anterior extensions [42]. The swim bladder is mainly an organ for controlling buoyancy [45], but in numerous fish species it is also involved in acoustic tasks such as enhancing hearing and producing sounds [5], [46]–[48]. Discussions of swim bladder specializations with respect to hearing enhancement must therefore take into account its multiple functions. Some bottom-dwelling fishes such as toadfishes have a slightly reduced swim bladder and utilize it mainly for sound production but not for hearing; others such as gobies, sculpins, or the cichlid *S. tinanti* have mostly or completely lost their swim bladders and show low auditory sensitivity, while bottom-dwelling loricariid and callichthyid catfishes have reduced their swim bladders to tiny encapsulated bubbles which still function as accessory hearing structure [39].

It is possible that the swim bladder morphology depends on its function as a sound production organ. The cichlid species presently studied do not possess swim bladder drumming muscles and have not yet been studied with regard to sound production. A study on the sound production mechanism in the cichlid *Oreochromis niloticus* [49] indicated that muscles situated ventro-lateral to the swim bladder seemed to be involved in sound production and were assumed to provoke a compression of the rib cage and the swim bladder. Thus, it remains to be investigated whether a similar mechanism in sound production may be present in the species used in our study and whether sound communication may have been a selective force in the evolution of the swim bladder diversity in cichlids.

Another set of important selective forces may have been ecological and ecoacoustical constraints. Rheophilic species such as *S. tinanti* [38] need to reduce their buoyancy in order to keep their position at the ground in fast-flowing waters. Besides this ecological factor we argue that ecoacoustical constraints influenced the evolution of swim bladder specializations in cichlids. Flowing waters are noisier than stagnant ones [50] and fish will not be able to utilize improved auditory sensitivities under such conditions [51]. Consequently we suggest that *P. polleni* and in particular *E. maculatus* live in rather stagnant and thus more quiet habitats and that they will be able to utilize their improved hearing abilities for detecting various sound sources at larger distances. This will include the detection of con-and heterospecifics as well as predators and prey [52].

We conclude that anterior swim bladder extensions in cichlids result in higher auditory sensitivity at frequencies above 0.3 kHz. Large swim bladders appear to increase the maximum frequency detectable while anterior extensions increase the auditory sensitivity in a midfrequency region at 0.5 and 1 kHz.
